# The Impact of Mechanical Recycling on Ligno-Cellulose Fibre Containing PLA Biocomposite

**DOI:** 10.3390/polym17060732

**Published:** 2025-03-11

**Authors:** Faizan Asad, Kirsi Immonen, Titta Kiiskinen, Atte Mikkelson, Essi Sarlin

**Affiliations:** 1 VTT Technical Research Centre of Finland, Visiokatu 4, 33101 Tampere, Finland; kirsi.immonen@vtt.fi; 2 VTT Technical Research Centre of Finland, Koivurannantie 1, 40400 Jyväskylä, Finland; 3 VTT Technical Research Centre of Finland, Tekniikantie 21, 02150 Espoo, Finland; 4 Materials Science and Environmental Engineering, Tampere University, Korkeakoulunkatu 7,33014 Tampere, Finland; essi.sarlin@tuni.fi

**Keywords:** biocomposite, softwood fibre, cellulose fibre, mechanical recycling, PLA, degradability

## Abstract

Biocomposites, made from biobased polymers with natural fibre reinforcement, offer a feasible path towards environment friendly and sustainable materials. However, biocomposites have struggled to attract ta market that is mostly dominated by conventional fossil-based polymers. To increase the cost efficiency and extend the lifespan of biocomposites, the effects of mechanical recycling on their properties should be thoroughly explored. While there has been some research on recycling natural fibre-reinforced biocomposites, limited attention has been paid to biocomposites reinforced with softwood fibre. This study investigates the impact of mechanical recycling on poly-lactic acid (PLA) biocomposites reinforced with bleached and unbleached softwood kraft pulp fibres at 15 wt% and 30 wt%. The results show that single-stage mechanical recycling improves Young’s modulus by 11–13% while maintaining impact strength. Tensile strength remains stable for biocomposites with 15 wt% fibre but decreases by 6–8% for with 30 wt% biocomposites. Recycling improves fibre dispersion by reducing agglomeration but leads to PLA degradation, which could potentially be mitigated by adding virgin polymer or chain extenders. These findings highlight the potential for reusing PLA-softwood fibre biocomposites while emphasizing the need for further research into multiple recycling cycles.

## 1. Introduction

As the demand for sustainable materials continues to grow, industries are increasingly turning to biodegradable solutions to address environmental concerns [1]. In this context, biocomposites composed of natural fibres and a biobased polymer present a viable option. Common examples of natural fibres used as reinforcement include jute, hemp, kenaf, flax, and softwood and hardwood pulp fibres [2,3,4]. Polylactic acid (PLA) is among the most widely used biobased polymers; PLA is a biodegradable polymer that is made from corn and sugarcane. It is used in a variety of applications, including medical devices, packaging, additive manufacturing, and different consumer goods. Although PLA offers mechanical properties comparable to fossil-based polymers, it is expensive compared to conventional polymers [5,6]. Furthermore, PLA is known for its brittleness, which has led to extensive research on combining PLA with various natural fibres. Combining natural fibres with PLA is an optimal approach, as it improves the mechanical properties of PLA [2,3,7,8] while preserving its sustainability.

While biodegradable biocomposites provide a sustainable solution that has apparent environmental benefits over fossil-based materials, their end-of-life (EoL) management poses challenges. Traditional recycling methods for fossil-based plastics might not be suitable for biodegradable biocomposites; thus, there is a need to investigate recycling strategies for biocomposites that could extend their lifecycle while maintaining their sustainability. There have been some studies performed that were based on the Life Cycle Assessment (LCA) of biocomposites. Haylock et al. in 2018 used LCA and techno-economic analysis for PLA composites with different organic and inorganic fillers. Two models were used to analyse recycling, incineration, landfill, landfill + methane extraction, and a base no-EoL option for biocomposites reinforced with glass, talc, flax fibre, hemp fibre, rice husks, and wood pulp fibre. The study concluded that organic-fibre-reinforced biocomposites lower the economic and environmental impact compared to fossil-based alternatives, particularly when considering end-of-life (EoL) strategies [9]. Uitterhaegen et al. in 2018 studied the recycling of PE and bio-based PE reinforced with coriander straws. Accelerated UV and hygrothermal ageing enhanced the flexural strength of Polypropylene (PP) biocomposites by 36%, while the native matrix experienced a 25% decrease. This ageing process reduced impact resistance in PP composites but improved it in bio-based polyethylene (BioPE) composites, highlighting the intricate relationship between polymer degradation and fibre/matrix interactions [10]. Beigbeder et al. in 2018 studied the EoL options with LCA, focusing on PLA/flax fibres and PP/wood flour biocomposites. The research considered recycling, composting, landfill, and incineration. The research concluded that mechanical recycling for both biocomposites has the lowest environmental impact among the studied EoL strategies [11]. In conclusion, mechanical recycling emerges as the most environmentally friendly end-of-life (EoL) strategy for biocomposites, although further research is needed to optimize these processes.

Mechanical recycling shows promising results in reducing waste and conservation of resources; however, for biodegradable biocomposites, it could cause the degradation of polymer and fibres. Several studies have investigated the effects of mechanical recycling on the properties of biocomposites. In 2017, Bhattacharjee et al. studied the feasibility of reprocessing natural-fibre-filled PLA composites. The oak wood flour PLA biocomposites were processed six times via extrusion and injection moulding with 30 and 50 wt% of filler. Polylactic acid grafted with maleic anhydride (PLA-g-MA, 3 wt%) was used with each recipe as a coupling agent. The research concluded that mechanical recycling caused an abrupt decrease in mechanical properties after the second cycle [12]. In 2019, Chaitanya et al. studied the mechanical recycling of PLA/sisal fibre biocomposites with eight extrusion cycles. The research showed that the biocomposite tensile strength reduced by about 21% up to the third cycle. The thermal and morphological analysis showed severe fibre and matrix degradation. The study concluded that after the third cycle, the mechanical recycling of PLA/sisal biocomposite is not recommended [13]. In 2024, Rao et al. studied recycling and degradation of PLA and bamboo-fibre-reinforced composites. In this study, the effect of different environmental conditions on the properties of biocomposites was investigated. Significant reductions in mechanical strength were observed, with tensile strength decreasing by up to 79.16% due to water exposure and soil degradation [14].

Some of the studies were performed on the mechanical recycling of biocomposites, where either the polymer or reinforcing agent was biodegradable. Nadali et al. in 2020 studied PVC and wood flour biocomposites for four extrusion cycles. The results concluded that bending strength and modulus of elasticity reduced over the cycles due to fibre-chain scission resulting from shear stress during processing, while impact strength almost remained constant [15]. In 2022, Zhou et al. found that MAPE as a compatibilizer improved the mechanical properties and dimensional stability of recycled high-filled wood fibre/polyethylene composites, although some degradation occurred during the recycling process. [16]. Finnerty et al. in 2023 investigated the mechanical recycling of PLA and basalt fibres and its effect on the mechanical properties of biocomposites. A conical twin extruder was used to perform six recycling cycles. The study concluded that, after the third cycle, the tensile strength significantly reduced due to a decrease in fibre length. The impact strength of the biocomposite did not significantly change after six cycles [17].

These studies indicate that the mechanical recycling of biocomposites should be thoroughly investigated to understand its impact on material properties and potential for reuse. To the best of our knowledge, no research has been conducted on the mechanical recycling of PLA matrices reinforced with softwood fibre and its effects on ligno-cellulose fibre and biocomposite properties. Previous studies on the mechanical recycling of PLA biocomposites using materials such as bamboo fibre, sisal fibre, and oak wood flour have shown varying impacts on mechanical properties and degradation behaviour. This highlights the novelty and importance of investigating softwood fibres, which may offer unique advantages in reducing waste and extending the lifecycle of biocomposites, even if some properties are compromised.

This paper aims to contribute new insights into the effects of mechanical recycling on PLA-based biocomposites reinforced with softwood fibres. In this research, we studied PLA with Bleached Softwood Kraft Pulp fibre (BSKPF) and Unbleached Softwood Kraft Pulp Fibre (UBSWPF). To reinforce the PLA, 15 and 30 weight percent of fibres were used. We explored the effects of one-stage mechanical recycling on fibres and the properties of the biocomposites.

## 2. Experimental

### 2.1. Materials

The polymer matrix was PLA Ingeo 3251D from NatureWorks LLC (Blair, NE, USA) with a weight-average molecular weight (Mw) of 103,196. Metsä Fibre’s Rauma mill in Finland supplied never dried bleached softwood pulp fibre (BSKPF) with 30% dry content and fibre pH about 6. Metsä Fibre’s Kemi pulp mill in Finland provided the never-dried unbleached pulp fibre (UBSKPF) with 30% dry content, which came straight from the alkaline pulping process with a pH > 7. Table 1 presents the composition of BSKPF and UBSKPF.

Prior to compounding, the fibres were dried and pelletized using a unique cone-shaped pelletizer, which was adapted from the KAHL pelletizer and further elaborated in a patent [18] that was produced at VTT.

### 2.2. PLA-BSKPF and PLA-UBSKPF Biocomposite Processing

The initial step involved drying the fibres overnight inside a vacuum oven at 50 °C. This drying process is important due to the hydrophilic and hygroscopic nature of cellulosic fibres. The objective was to minimize moisture content within the fibres, as excessive moisture could adversely impact the processing of biocomposites and lead to the hydrolytic degradation of PLA. The PLA was dried in a vacuum oven for about 2 h at a temperature of 50 °C.

For the compounding of each recipe listed in Table 2, a co-rotation twin screw extruder (Berstorff ZE 23 × 33D; Berstorff GmbH, Hannover, Germany) equipped with eight temperature zones was employed. The components were introduced into the twin screw extruder using a gravimetric feeder while maintaining a temperature profile of 165–185 °C. The extruder operated at 150–175 revolutions per minute (rpm).

Subsequently, both PLA-BSKPF and PLA-UBSKPF biocomposites underwent drying in a vacuum oven set at 50 °C for about 14 h. Following this drying process, the biocomposites were subjected to injection moulding using a Battenfeld injection moulding machine (SmartPower 25–400 t, Wittmann Battenfeld GmbH, Kottingbrunn, Austria) with a temperature profile of 195–190–190–190 °C across its four temperature zones. The first injection cycle employed a mould temperature of 32 °C.

Next, the PLA-BSKPF and PLA-UBSKPF biocomposites were ground with a Rapid type 1514 grinder (Rapid Granulator, Bredaryd, Sweden) with a 4 mm mesh and subsequently dried in a vacuum oven at 50 °C overnight. This process step simulates the recycling process. The dried and ground biocomposites were then once again subjected to injection moulding, under conditions similar to the initial injection process except a mould temperature of 35 °C. The materials after recycling are referred with prefix ‘r’ (e.g., rPLA 15B).

**Figure 1:**
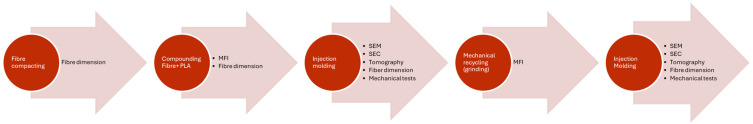
Unit process flow of PLA-BSKPF and PLA-UBSKPF biocomposites.

Figure 1 illustrates the process flow for the manufacturing and characterization of PLA-BSKPF and PLA-UBSKPF biocomposites.

### 2.3. Characterization

Melt flow index (MFI) measurements were conducted after the compounding process and after the grinding of the biocomposite. Tensile strength, Charpy impact tests, scanning electron microscopy (SEM), differential scanning calorimetry (DSC), and size exclusion chromatography (SEC) analyses were carried out following the first and second injection-moulding processes. Cellulose fibre characterization measurements were made for compounds after compounding, the first injection moulding and the second injection moulding process steps.

#### 2.3.1. Melt Flow Index

The MFI (melt flow index) is the amount of molten material that flows via a typical die’s capillary tube in a span of ten minutes. In accordance with ISO 1133-1 (2011) [19], the MFI of the biocomposites was assessed at a temperature of 190 °C and a load of 2.16 kg using RAY-RAN Melt Flow Indexer, Model 3A (Industrial Physics, Theme, UK).

#### 2.3.2. Scanning Electron Microscope

The SEM was used to analyse the injection-moulded biocomposite samples’ morphology. A cross-sectional analysis of the test bars was conducted. A JEOL JSM T100 SEM from JEOL Ltd. (Tokyo, Japan) was used for this purpose. Fractured injection-moulded samples from the impact tests were used. A thin layer of gold, approximately 5 nm in thickness, was sputtered over the sample surface to avoid surface charge accumulation.

#### 2.3.3. Size Exclusion Chromatography

Molar mass measurements were conducted using size exclusion chromatography (SEC) with chloroform (Sigma Aldrich, purity ≥ 99.8%) as the eluent. The samples were ground with a Wiley Mill model 3 (Arthur H. Thomas Co., Philadelphia, PA, USA) before being dissolved in chloroform overnight at a concentration of 20 mg/mL. All samples were filtered through a 0.45 µm filter prior to measurement.

SEC measurements were carried out in chloroform eluent (0.6 mL/min, 30 °C) using Styragel HR 4 and 3 columns along with a pre-column. Elution curves were detected with the Waters 2414 Refractive Index Detector (Waters Corporation, Milford, CT, USA). Molar mass distributions (MMDs) were calculated using Waters Empower 3 software 7.50.00.00, calibrated against eight PS standards (1260–1,030,000 g/mol). Degradation indices for the processed were calculated from the data by the following equation:

DI = (1 − Mw_PLA_/Mw_biocomposite)_ × 100%

DI is the degradation index (expressed as a percentage).

Mw_PLA_ is the average molecular weight of PLA.

Mw_biocomposite_ is the average molecular weight of the polymer matrix of the biocomposite.

#### 2.3.4. Microtomography

A desktop microtomography scanner (DeskTom 130, RX Solutions, Chavanod, France) was used to study the cross-sectional structure of the biocomposites. Pieces measuring 5 mm × 5 mm × 10 mm were cut from the middle part of the test bars. The pieces were imaged using 40 kV acceleration voltage and 4 W electron beam power. The voxel size was 3.4 µm, and a total of 1440 projection images were collected over a 360-degree rotation. The exposure time was 1.43 s for each frame, and three frames were averaged for each projection image to enhance the signal-to-noise ratio. This resulted in a total imaging time of approximately 1 h and 43 min for each sample.

Quantitative analysis was performed for porosity and fibre agglomerate fraction from µCT images. First, the intensity level of voids in the images was determined; then, the images were cropped to contain only the sample. The cropped images were denoised using Gaussian filtering with σ = 0.5 voxels. The voids were detected by thresholding, and the porosity was calculated as the fraction of void voxels. Cupping artefacts were removed by high pass filtering by removing the slowly changing background determined via a Gaussian filter with σ = 50 voxels. To avoid artefacts due to the pores, the pores were filled by the mean value of the image prior to calculating the slowly changing background.

The threshold level for detecting fibre voxels was chosen to yield the expected volume fraction for the fibres. A binarized image with true values for the fibre voxels was obtained by thresholding. To detect the agglomerates, the binarized image was converted to 8-bit unsigned integer format and blurred by a Gaussian filter with σ = 4 voxels (36 µm) to determine local fibre density. Voxels with fibre density above 50% are considered agglomerate regions. Agglomerate fraction is given by the fraction of agglomerate voxels of all fibre voxels. The SciPy and Scikit-Image libraries were used to implement the Python 3.12 algorithm [20,21].

#### 2.3.5. Differential Scanning Chromatography (DSC)

The analysis of both pristine PLA and biocomposite samples involved the use of a NETZSCH DSC 204F1 Phoenix 240-12-0287-L apparatus from NEZSCH GmbH (Selb, Germany). In this analysis, about 5–6 mg of material was subjected to a predetermined thermal profile. The initial heating cycle employed a ramp rate of 10 °C/min, starting at 0 °C and gradually increasing to 200 °C. Subsequently, the samples were cooled to 0 °C at a rate of 10 °C/min.

To calculate the degree of crystallization, the following formula was applied:

Degree of Crystallization (%) = (ΔHm − ΔHc)/ΔHf × 100

where ΔHf is the enthalpy of fusion of 100% crystalline PLA at equilibrium melting point (93.6 J/g), ΔHm is the melting enthalpy, and ΔHc is the cold crystallization enthalpy [22].

#### 2.3.6. Fibre Analysis

For the determination of the fibre dimensions, the fibre fraction of compounded and injection-moulded samples was Soxhlet-extracted. PLA was extracted using hot chloroform and for min 48 h. A small amount of composite sample was placed into the Soxhlet thimble and continuously fluxed with a hot solution and in a cool solution during night-time. The remaining kraft pulp fibre fraction was collected, and the fibre lengths were determined using an L&W Fibre Tester Plus–Code 912+ (Lorenzen & Wettre, Kista, Sweden). Some 0.23–0.30 g of each sample was soaked in 500 mL of deionized water, and they were dispersed using a high shear laboratory blender, IKA Ultra-Turrax T18 (IKA-Werke GmbH, Staufen im Breisgau, Germany) for five minutes.

After dispersing, the samples were left in deionized water overnight. The next day, the samples were further mixed in five litres of water with an impeller for 10 min. Then, 50 mL of dispersed sample was pipetted into the analyser, and the fibre analysis was performed with two parallel measurements. Fibre length results were reported according to TAPPI standard method T271 [23].

#### 2.3.7. Charpy Impact Strength

ISO-179-2 (1997) [24] standard was followed while testing for the Charpy impact strengths, which were measured on an impact strength device, Charpy Ceast Resil 5.5 (CEAST S.p.a., Torino, Italy) for ten unnotched samples.

#### 2.3.8. Tensile Strength

A five-day conditioning period at 23 °C and 50% relative humidity was conducted on injection-moulded samples. The tensile test was performed using an Instron 4505 Universal Tensile Tester (Instron, Norwood, MA, USA). The crosshead speed was set to 5 mm/min, and the load cell force was 10 kN. The ISO-527-2 (2012) [25] standard was followed, and the strain was measured with an Instron 2665 Series High Resolution Digital Automated Extensometer. Six samples of each biocomposite were tested, and standard deviations and averages were calculated for the tensile strength and Young’s modulus.

As per ISO-527-2 (2012) specifications, the tensile samples were required to have a thickness within the range of 4 ± 0.2 mm, a width of 10 ± 0.2 mm, and a gauge length of 170 mm. These dimensional tolerances were monitored to ensure consistency and precision in the measurement of mechanical properties.

## 3. Results and Discussion

### 3.1. Melt Flow Index

**Figure 2:**
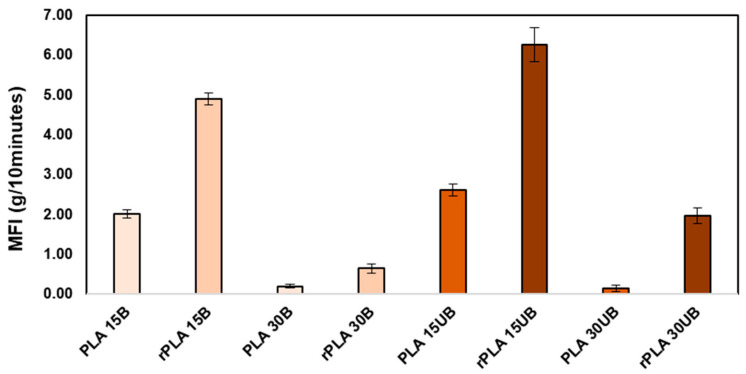
The melt flow index of neat PLA, PLA-BSKPF, and PLA-UBSKPF biocomposites and their respective recycled compounds before injection moulding.

Figure 2 shows the MFI of neat PLA, PLA-BSKPF, and PLA-UBSKPF biocomposites and their respective recycled biocomposites before injection moulding. The MFI of PLA is about 32.2 g/10 min at 190 °C and a load of 2.16 kg; it is absent from the graph since there is quite a major difference in scale compared to the MFI of biocomposites. The increase in viscosity of PLA-BSKPF and PLA-UBSKPF biocomposites due to fibre addition resulted in a decrease in melt flowability and, hence, a decrease in the MFI. The decrease in the MFI with an increase in the fibre percentage is consistent with the literature review [26]. Moreover, the significant increase in the MFI of PLA-UBSKPF is attributed to the plasticizing effect of lignin [27,28], which reduces intermolecular interactions and improves chain mobility, particularly after recycling.

After mechanical recycling, the MFI of PLA-BSKPF increased by about 143% for PLA 15B and 237% for PLA 30B. Similarly, the MFI of PLA-UBSKPF biocomposites increased after mechanical recycling by about 140% for PLA 15UB and 1307% for PLA 30UB. The increase in MFI is due to polymer chain scission during grinding and injection moulding, which reduces molecular weight and improves melt flowability. This aligns with the literature review, which demonstrates that the MFI of neat PLA increases after mechanical recycling [29].

### 3.2. Scanning Electron Microscope

**Figure 3:**
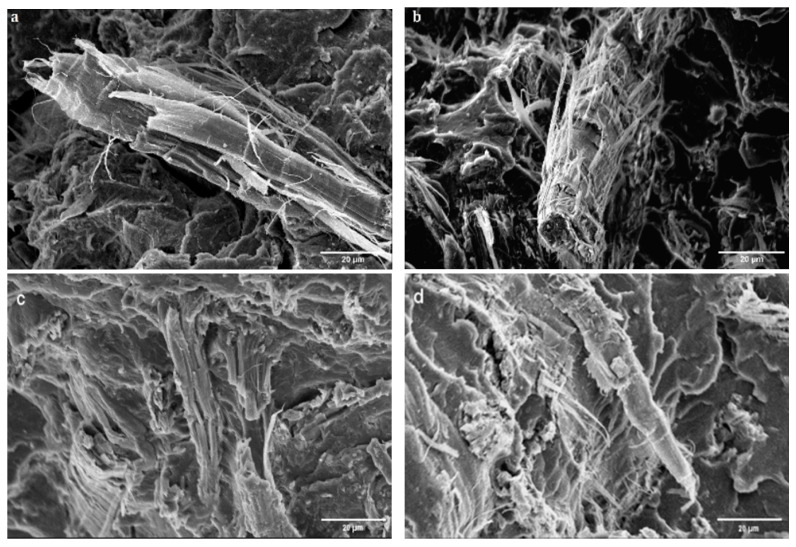
SEM images of (**a**) PLA30B, (**b**) PLA30UB, (**c**) rPLA30B, and (**d**) rPLA30UB.

Figure 3 presents the SEM images of PLA 30B, rPLA 30B, PLA 30UB, and rPLA 30UB. PLA 30B and PLA 30UB in Figure 3a,b show that there is fibrillation in both bleached and unbleached fibre-based biocomposites. However, there is more fibrillation and opening up of fibre in PLA 30B; this can be attributed to a lack of lignin in BSKPF that makes the fibre more susceptible to cellulose fibril tearing [30,31]. Lignin provides inter-fibre bonding and prevents the opening of fibres.

rPLA 30B in Figure 3c shows that recycling caused the breaking and opening up of bleached fibre, while rPLA 30UB in Figure 3d shows that, after recycling, the extent of fibrillation and fibre breaking is less compared to rPLA 30B.

### 3.3. Size Exclusion Chromatography

**Figure 4:**
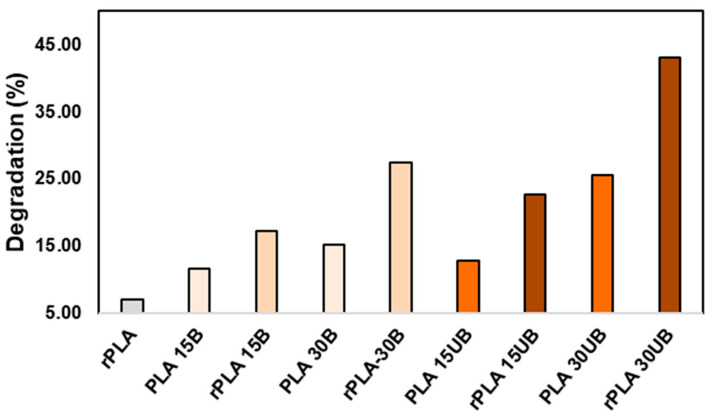
The percentage of degradation of neat PLA, PLA-BSKPF, and PLA-UBSKPF after injection moulding and mechanical recycling.

Figure 4 shows the degradation percentage of PLA, PLA-BSKPF, and PLA-UBSKPF biocomposites after injection moulding and mechanical recycling. The type and quantity of fibre notably affect the degradation rate of biocomposites. The biocomposites with higher fibre content showed higher polymer degradation. The degradation is caused by mechanical and thermal stresses, leading to polymer chain scission, which the recycling process further exacerbates. This is in line with the literature, which shows that the recycling of PLA and PLA biocomposites causes degradation of PLA matrix [17,32,33].

Moreover, in PLA-UBSKPF biocomposites, the degradation rate is higher than in PLA-BSKPF biocomposites before and after mechanical recycling. PLA degrades more in basic pH, since the UBSKPF comes from an alkaline pulping process, which is why there is more polymer degradation in PLA-UBSKPF [34].

### 3.4. Microtomography (µCT)

**Figure 5:**
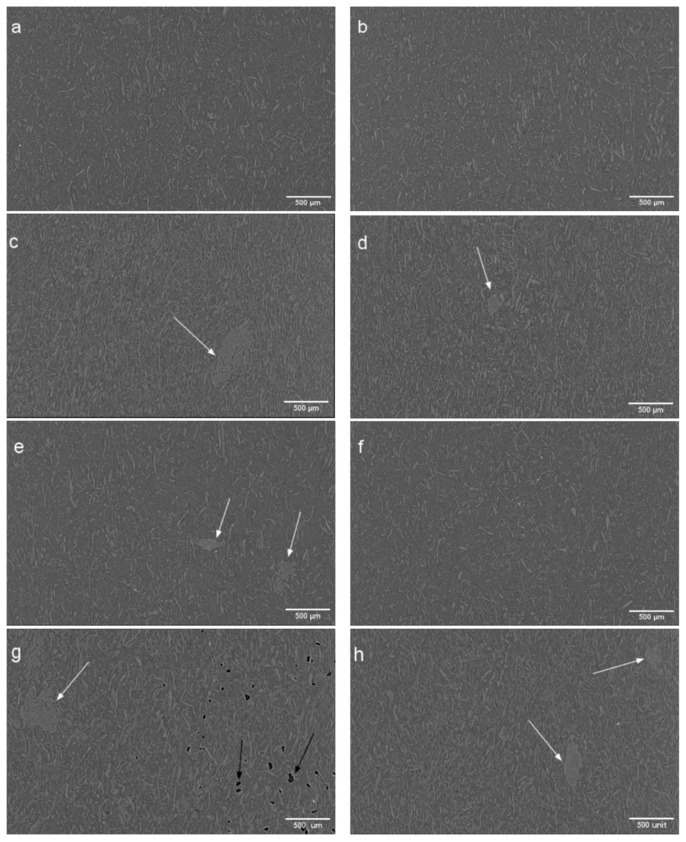
The X-ray tomography images of PLA 15B (**a**), rPLA 15B (**b**), PLA 30B (**c**), rPLA 30B (**d**), PLA 15UB (**e**), rPLA 15UB (**f**), PLA 30UB (**g**), and rPLA 30UB (**h**); white arrows indicate the fibre agglomerates, and black arrows show voids.

Figure 5 shows the X-ray tomography images of PLA-BSKPF and PLA-UBSKPF biocomposites. The tomographic images show that the fibres are well dispersed in the PLA matrix, and the lower-fibre-fraction biocomposites have smaller-sized fibre agglomerates, as shown in Figure 5a,e.

**Figure 6:**
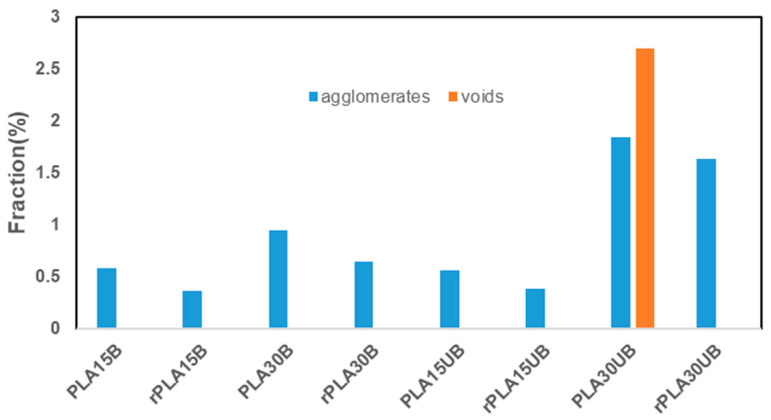
Agglomerates and voids fraction of PLA-BSKPF and PLA-UBSKPF biocomposites.

Moreover, there is void formation in PLA 30UB, as shown in Figure 5g. This is also visible in Figure 6, which shows the agglomerates and voids fraction of PLA-BSKPF and PLA-UBSKPF biocomposites. As PLA 30UB is the only sample with void formation, it is possible that during processing, it was contaminated with some foreign material or moisture, which may have led to the formation of voids in the sample. However, it could also be related to its lowest MFI among all the samples, as shown in Figure 2. The lowest MFI means that PLA 30UB has the highest melt viscosity that restricts the smooth flow polymer and hence causes void formation. However, rPLA 30UB (Figure 5h and Figure 6) had no voids, possibly due to increased degradation during recycling, as shown in Figure 4. The polymer degradation decreases molecular weight and hence results in higher MFI and improved melt flowing properties that lead to no void formation.

Figure 5 and Figure 6 show that the agglomerate fraction is higher in high-fibre PLA biocomposites, and that recycling decreased the size of agglomerates and prevented voids. Upon recycling, the agglomerate content was reduced by 37% in PLA 15B and 31% in PLA 30B. Similarly, reductions of 31% and 11% were observed in PLA 15UB and PLA 30UB, respectively.

### 3.5. Differential Scanning Calorimetry

Table 3 shows the DSC results for PLA, PLA-BSKPF, and PLA-UBSKPF biocomposites and their respective recycled biocomposites. In general, mechanical recycling of PLA-BSKPF and PLA-UBSKPF biocomposites caused a reduction in the degree of crystallization. This reduction could be because of the thermal and physical degradation of biocomposites during grinding and injection-moulding processes. PLA-BSKPF and PLA-UBSKPF biocomposites show a lower degree of crystallization in the higher fibre concentrations, as fibres in an agglomerate have a lower specific surface area compared to dispersed fibres, resulting in fewer nucleation sites for crystallization. Moreover, PLA 30UB has the lowest degree of crystallization among all biocomposites. The lowest degree of crystallization in PLA 30UB results in the formation of a significant number of voids, as shown in Figure 5. A lower degree of crystallization reduces the material’s density, which can lead to the formation of voids [35]. After mechanical recycling, the degree of crystallization of PLA 30UB increases due to the absence of voids and the reduction in the size of agglomerates. This creates more nucleation sites, which facilitate the crystallization process by promoting better chain alignment and packing.

In general, recycling of PLA, PLA-BSKPF, and PLA-UBSKPF biocomposites slightly reduced the Tg and Tc values, except for PLA 30UB. The slight decrease in Tg and Tc values after recycling is due to polymer degradation. During the recycling process, the polymer undergoes mechanical and thermal stresses that cause chain scission, and a smaller chain requires less energy to move and reorganise, which results in lower Tg and Tc values. The decrease in Tg and Tc values after recycling aligns with the literature [13].

### 3.6. Fibre Behaviour

**Figure 7:**
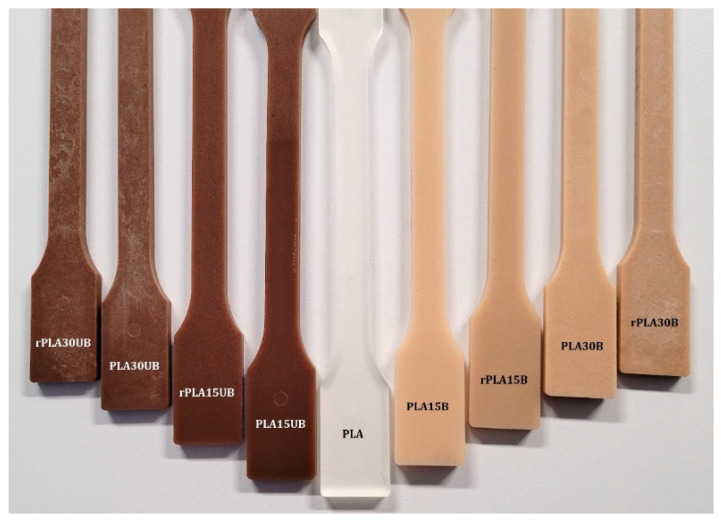
Visual outlook of injection-moulded samples.

Figure 7 shows the visual representation of injection-moulded samples. On the left is PLA-UBSKFP with dark brown colour, in the middle is PLA, which is translucent, and on the right is PLA-BSKPF, with a light-brown colour.

#### 3.6.1. Average Fibre Length

**Figure 8:**
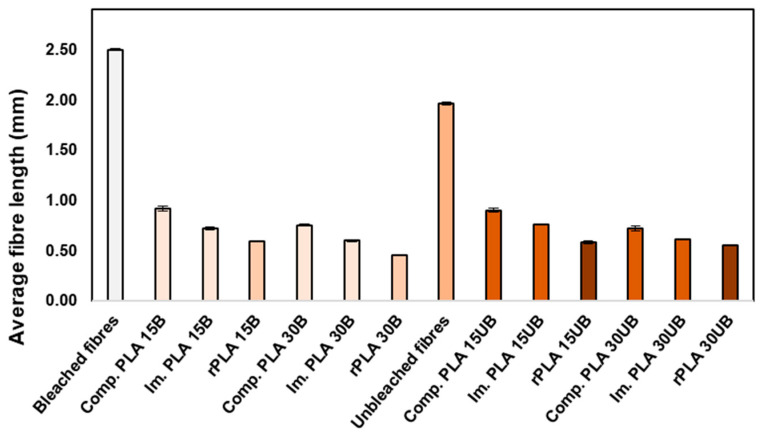
The average fibre length of BSKPF and UBSKPF and average fibre length in PLA-BSKPF and PLA-UBSKPF after compounding, injection moulding, and mechanical recycling.

In Figure 8 are the average fibre lengths of pristine BSKPF and UBSKPFs and the average fibre lengths in PLA-BSKPF and PLA-UBSKPF biocomposites after compounding, injection moulding, and mechanical recycling. The prefix “Comp.” represents biocomposites after compounding, and “Im.” represents biocomposites after injection moulding.

During compounding, the average fibre length decreased considerably in both PLA-BSKPF and PLA-UBSKPF biocomposites. During compounding, the fibres experienced mechanical shear forces in between the screws of the twin screw extruder. Furthermore, the compounding temperature (195 °C) softens the fibres and promotes fibre breaking. The average fibre length further decreased during the injection moulding and the mechanical recycling. For PLA-BSKPF, the average fibre length of rPLA 30B decreased to about 18% compared to bleached fibres, while in PLA-UBSKPF, the average fibre length reduced to about 28% compared to unbleached fibres. The decrease in fibre length after mechanical recycling is in line with the literature [13]. For higher fibre biocomposites, the decrease in average fibre length is higher because high fibre content increase fibre–fibre and fibre–machine interactions, which amplify shear forces and lead to more breakage of the fibres, reducing the average fibre length.

It should be noted that the method used for fibre analysis employed a high-shear laboratory blender for the dispersion of samples that might influence the change in average fibre length, average fibre width, and percentage of mean fibre fines of the PLA-BSKPF and PLA-UBSKPF biocomposites.

#### 3.6.2. Average Fibre Width

**Figure 9:**
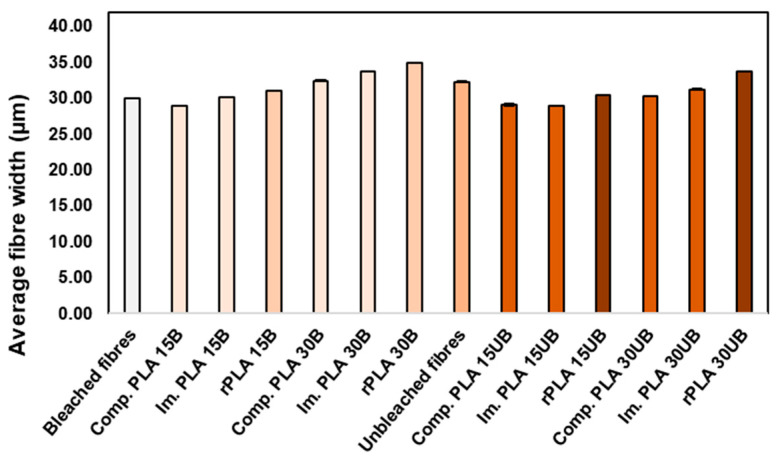
The average fibre width of BSKPF and UBSKPF and the average fibre width in PLA-BSKPF and PLA-UBSKPF after compounding, injection moulding, and mechanical recycling.

In Figure 9 are the average fibre width of BSKPF and UBSKPF and the average fibre width in PLA-BSKPF and PLA-UBSKPF biocomposites after compounding, injection moulding, and mechanical recycling. The average fibre width in the PLA-BSKPF biocomposites has slightly increased after each process step except Comp. PLA 15B. The increase in the average fibre width of PLA-BSKPF biocomposites is because of fibrillation and opening of bleached fibres, as supported by SEM images in Figure 3. For comparison, the average fibre width of PLA 15B after recycling increased by 3.2% compared to the average fibre width of bleach fibre. In contrast, the average fibre width of PLA 15UB after recycling decreased by 5.9% compared to the average fibre width of unbleached fibre.

In general, the average fibre width of the PLA-UBSKPF biocomposites is less than the average fibre length of the unbleached fibre. This aligns with the literature, which reports that the fibre width decreases during biocomposite processing [35]. The decrease in fibre width can be attributed to the compression and shear forces applied during processing. However, the average fibre width of PLA-BSKPF slightly increased after the recycling process. This is because the bleached fibre broke and opened up, as seen in the SEM in Figure 3a.

#### 3.6.3. Mean Fibre Fines Percentage

**Figure 10:**
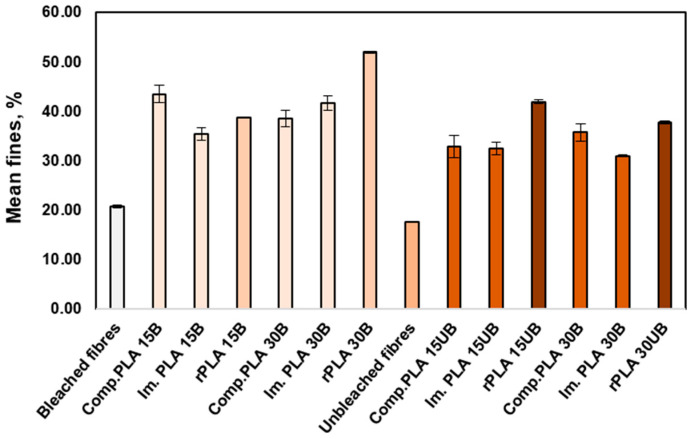
Mean fines percent of BSKPF and UBSKPF and average fibre width in PLA-BSKPF and PLA-UBSKPF after compounding, injection moulding, and mechanical recycling.

In Figure 10, the graph shows the mean fine percentage in BSKPF, UBSKPF, PLA-BSKPF, and PLA-UBSKPF biocomposites after compounding, injection moulding, and mechanical recycling. The fines originate from fibre cutting during compounding, injection moulding, and recycling processes.

PLA-BSKPF biocomposites have a relatively high mean fines percentage compared to PLA-UBSKPF; this is related to the more fibrillation and hence more cutting of fibres in PLA-BSKPF compared to PLA-UBSKPF, as shown in SEM in Figure 3a.

The mechanical recycling increased the number of fines for each biocomposite. The recycling involved the biocomposites passing through additional unit processes, which include grinding, extrusion, and injection moulding, which resulted in further fibre cutting and consequently increased the percentage of the mean fine.

### 3.7. Mechanical Properties

#### 3.7.1. Charpy Impact Strength

**Figure 11:**
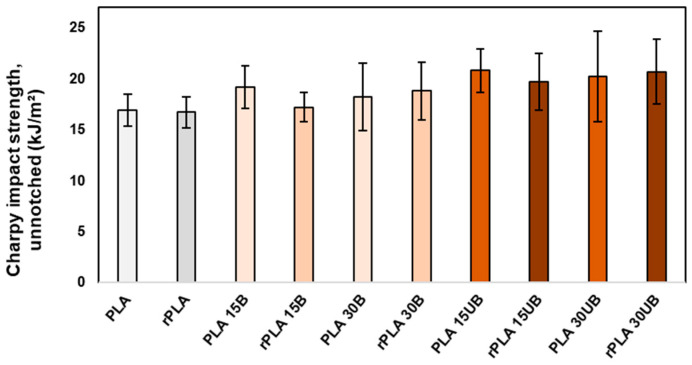
The Charpy impact strength of neat PLA, PLA-BSKPF and PLA-UBSKPF after injection moulding and mechanical recycling.

Figure 11 presents the impact strength of neat PLA, PLA-BSKPF, and PLA-UBSKPF after injection moulding and mechanical recycling. The impact strength of neat PLA remains nearly unchanged after mechanical recycling.

PLA-BSKPF and PLA-UBSKPF biocomposites showed improved impact strength compared to neat PLA. PLA-UBSKPF showed slightly better results compared to PLA-BSKPF. The presence of lignin in PLA-UBSKPF biocomposites made them tougher than PLA-BSKP, enabling them to absorb more impact energy. Furthermore, the SEM result in Figure 3 showed that PLA-UBSKPF biocomposites retain better fibre structure, which allows them to absorb more impact energy than PLA-BSKPF.

After recycling, the impact strength of PLA-BSKPF and PLA-UBSKPF biocomposites remains nearly the same for both PLA 15A and PLA 15UB. Similarly, the impact strength of PLA 30B and PLA 30UB remains stable, even with polymer degradation, as shown in Figure 4. This stability in impact strength after recycling is likely due to improved fibre dispersion and the breakdown of agglomerates, as illustrated in Figure 6. Furthermore, the reduction in average fibre length after mechanical recycling (as shown in Figure 8) enhances fibre dispersion, leading to better stress transfer and preservation of impact strength. In the literature, it has been reported that the impact strength of PLA–sisal biocomposites decreases progressively over the course of eight mechanical cycles, primarily due to the degradation of the PLA matrix [13].

#### 3.7.2. Young’s Modulus 

**Figure 12:**
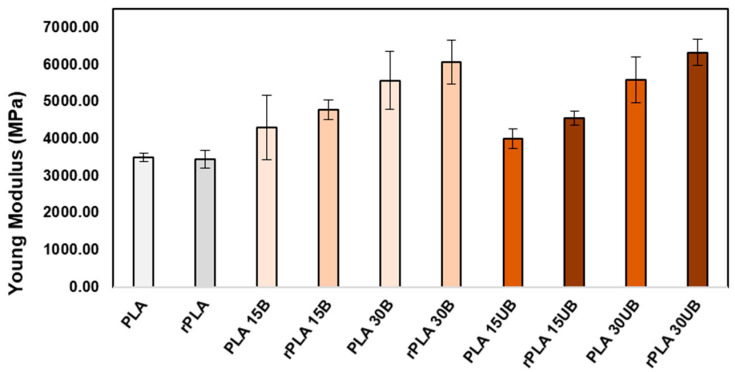
The Young’s modulus of neat PLA, PLA-BSKPF, and PLA-UBSKPF after injection moulding and mechanical recycling.

Figure 12 presents the Young’s modulus of neat PLA, PLA-BSKPF, and PLA-UBSKPF after injection moulding and mechanical recycling. The addition of BSKPF and UBSKPF improved the modulus of PLA-BSKPF and PLA-UBSKPF biocomposites.

Furthermore, the recycling caused an improvement in the modulus of about 8–13% for each PLA-BSKPF and PLA-UBSKPF biocomposite. This can be attributed to the breakdown of large-size agglomerates, as seen in Figure 6. Reduced void formation after recycling, as shown in Figure 5 and Figure 6, improves density and stiffness, contributing to the increase in Young’s modulus. Additionally, shorter average fibre lengths post-recycling as shown in Figure 8, improve dispersion and lead to better stiffness. Although mechanical recycling caused degradation as shown in Figure 4, which may lower molecular weight through polymer chain scission; however, it improves fibre distribution and reduces porosity, supporting the modulus improvement. The increase in Young’s modulus after recycling of PLA biocomposites with biobased fibres is consistent with the findings reported in the literature [36].

#### 3.7.3. Tensile Strength

**Figure 13:**
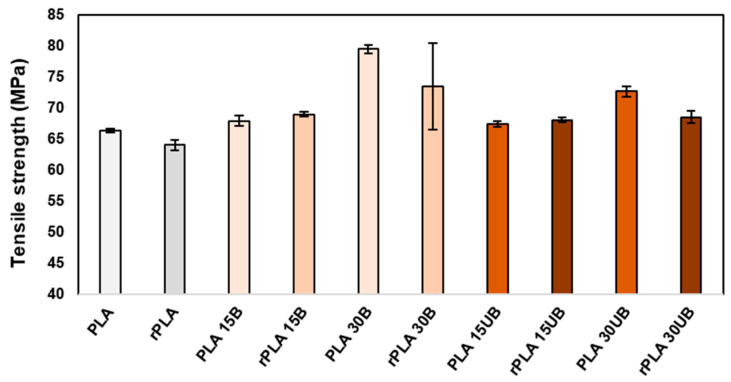
The tensile strength of neat PLA, PLA-BSKPF, and PLA-UBSKPF after injection moulding and mechanical recycling.

Figure 13 shows the tensile results of neat PLA, PLA-BSKPF, and PLA-UBSKPF biocomposites after injection moulding and mechanical recycling. PLA 30B has the highest tensile strength. PLA-BSKPF biocomposites have better tensile strength than PLA-UBSKPF biocomposites, which is related to more fibrillation in PLA-BSKPF compared to PLA-UBSKPF. Furthermore, higher polymer degradation in PLA-UBSKPF affects the tensile strength, as shown in the SEC results in Figure 4.

After the mechanical recycling of PLA 15B and PLA15UB, the tensile strength remained consistent, indicating an improved fibre dispersion by a reduction in agglomerates, as shown in Figure 6. Furthermore, fibrillation mitigated the effects of PLA degradation in PLA 15B and PLA 15UB. However, the tensile strengths of PLA 30B and PLA 30UB experienced a decrease of about 8 and 6%.

The decrease in the tensile strength of higher fibre biocomposites is a result of higher degradation values, as seen in SEC results in Figure 4. Moreover, rPLA 30B and rPLA 30UB have the lowest average fibre length (Figure 8), and the decrease in average fibre length also reduces the reinforcement effect of the fibres, leading to a less efficient stress transfer between matrix and fibres. The decrease in the tensile strength of PLA after mechanical recycling is in line with the literature review; for both neat PLA and PLA biocomposites, the tensile strength decreases after mechanical recycling [17,29].

**Figure 14:**
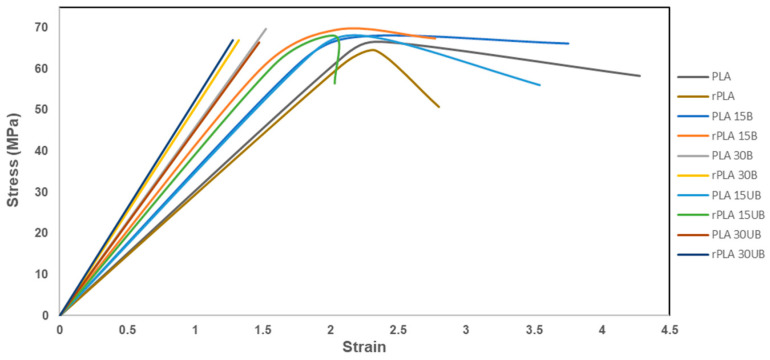
Stress–strain curves of neat PLA, PLA-BSKPF, and PLA-UBSKPF after injection moulding and mechanical recycling.

Figure 14 shows that the addition of fibres improved the tensile strength and yield strength of PLA biocomposites; however, it also reduces their ductility, especially for the 30% fibre biocomposites, which exhibit significantly higher stiffness and a predominantly elastic behaviour with minimal plastic deformation before breaking. Mechanical recycling further reduces both the tensile strength and ductility of the biocomposites.

Mechanical recycling of PLA biocomposites leads to polymer degradation, reduced fibre length, and weakened fibre–matrix interactions, resulting in a decline in tensile strength for high-fibre-content (30 wt%) PLA biocomposites. However, PLA with 15 wt% fibre content maintained consistent tensile strength, demonstrating the reinforcement effect of cellulose. PLA-UBSKPF exhibited higher degradation rates compared to PLA-BSKPF; however, both PLA-BSKPF and PLA-UBSKPF maintained their modulus and impact strength after a single stage of mechanical recycling. These findings highlight the potential for improving the recyclability and lifespan of PLA biocomposites.

The mechanical properties and recyclability of PLA biocomposites highlight their potential in industries prioritizing sustainability, lightweight materials, and biodegradability. PLA-BSKPF and PLA-UBSKPF biocomposites, with improved stiffness, are well-suited for automotive and packaging applications, offering weight reduction for improved fuel efficiency and reduced plastic waste.

## 4. Conclusions

In this research, we studied the effects of mechanical recycling on PLA biocomposites reinforced with 15% and 30% weight percent of bleached and unbleached softwood fibres. The biocomposites were compounded with PLA and compacted fibres, followed by injection moulding. The injection-moulded samples were then ground and then were injection-moulded again. Extensive characterization methods were employed to analyse the PLA biocomposites both before and after mechanical recycling.

- Single-stage mechanical recycling of PLA–cellulosic fibre composites does not compromise the impact strength of the biocomposites and improves the Young’s modulus by 11–13%.
- For fibre content of 15 wt%, the tensile properties of the PLA biocomposite material remained intact, while for PLA with 30 wt% fibre, the tensile strength decreased by up to about 6–8% after mechanical recycling.
- The mechanical recycling of PLA–cellulose fibre composites improved fibre dispersion by reducing agglomeration by 11–37%.
- The mechanical recycling of PLA–cellulose fibre composites resulted in a notable degradation of 5–23% in PLA.
- The effect of PLA degradation can be compensated by adding a virgin polymer during recycling or using chain extenders, which is a typical method to maintain polymer chain length during recycling.
- Further research is needed on multiple mechanical recycling steps to evaluate the impact of mechanical recycling on PLA softwood fibre biocomposites.

## Figures and Tables

**Table 1:** BSKPF and UBSKPF composition according to the manufacturer.

Sample	Cellulose (%)	Hemicellulose (%)	Lignin (%)
BSKPF	85	15	-
UBSKPF	77	16	7

**Table 2:** The parameters of compounding of PLA-BSKPF and PLA-UBSKPF biocomposites.

Composition	Sample Code	Screw Speed (rpm)	Zone Temperature (°C)
PLA 85 wt.% + BSKPF 15 wt.%	PLA 15B	150–175	165–175–185–190–190–190–190–185
PLA 70 wt.% + BSKPF 30 wt.%	PLA 30B	150–175	165–175–185–190–190–190–190–185
PLA 85 wt.% + UBSKPF 15 wt.%	PLA 15UB	150–175	165–175–185–190–190–190–190–185
PLA 70 wt.% + UBSKPF 30 wt.%	PLA 30UB	150–175	165–175–185–190–190–190–190–185

**Table 3:** The DSC of PLA-BSKPF and PLA-UBSKPF biocomposites and their one-stage-recycled compounds.

Sample	Tg (°C)	Tc (°C)	ΔHc (J/g)	Tm (°C)	ΔHm (J/g)	Degree of Crystallization (%)
PLA	61.1	100.9	28.6	170.4	39.6	11.8
rPLA	60.3	100.3	29.9	169.8	37.9	8.5
PLA 15B	62.5	98.9	24.3	168.4	48.0	25.4
rPLA 15B	62.4	96.4	17.7	168.6	40.6	24.6
PLA 30B	62.7	95.5	12.6	168.5	36.2	25.3
rPLA 30B	62.3	93.4	18.8	168.0	36.1	18.5
PLA 15UB	62.2	99.1	19.6	168.1	41.4	23.4
rPLA 15UB	61.4	99.1	18.7	168.5	35.3	17.8
PLA 30UB	60.0	98.1	12.6	168.7	24.3	14.2
rPLA 30UB	61.1	96.0	18.1	167.9	39.8	23.2

## Data Availability

The original contributions presented in the study are included in the article, further inquiries can be directed to the corresponding author.
